# Estimating the Nucleation Ability of Various Surfaces Towards Isotactic Polypropylene via Light Intensity Induction Time Measurements

**DOI:** 10.3390/e21111068

**Published:** 2019-10-31

**Authors:** Enrico Carmeli, Bao Wang, Paolo Moretti, Davide Tranchida, Dario Cavallo

**Affiliations:** 1 Dipartimento di Chimica e Chimica Industriale, Università degli studi di Genova, via Dodecaneso 31, 16146 Genova, Italy; enrico.carmeli@edu.unige.it (E.C.); bao.wang@edu.unige.it (B.W.); paolo.moretti@unige.it (P.M.); 2 Borealis Polyolefine GmbH, Innovation Headquarters, St. Peterstrasse 25, 4021 Linz, Austria; davide.tranchida@borealisgroup.com

**Keywords:** crystal nucleation, polypropylene, nucleating agents, induction time, heterogeneous nucleation

## Abstract

Crystallization of isotactic polypropylene (iPP) at the interface with crystalline films of two commercially employed nucleating agents (sodium benzoate (NaBz) and sodium 2,2’-methylene *bis*-(4,6-di-*tert*-butylphenyl)phosphate (NA-11)) and with a glass fiber (GF) was investigated using a polarized optical microscope. The analysis of the light intensity evolution during the crystallization process enabled the successful estimation of the time at which the crystal growth began, i.e., the induction time (*t_i_*), at various crystallization temperatures. Meaningful differences in the *t_i_* values were observed between the investigated systems. Moreover, the *t_i_* data have been analyzed according to different nucleation models proposed in the literature, which consider either the time to form the first crystalline layer in contact with the substrate or the time required to grow a cluster of critical size. It has been found that the two models are applicable in different temperature ranges depending on the efficiency of the given substrate. Therefore, in order to obtain the value of the surface free energy difference function, Δ*σ*, which is directly related to the nucleation energy barrier and useful for the definition of a universal nucleating efficiency scale, a model that considers both the above-mentioned times was fitted to the overall data. The values of Δ*σ* for the nucleation of iPP on the surface of the different substrates are thus obtained and discussed in the framework of the literature results.

## 1. Introduction

In modern polymer technology, the use of additives (nucleating agents, pigments, fillers, etc.) has a very important role because it allows for tuning and improvement of the properties of materials. In the case of nucleation, an extremely small number of foreign particles may be responsible for heterogeneous nucleation, in which the crystalline embryo can form on solid surfaces already present in the polymer melt. Several different types of heterogeneity (extraneous solids, cavities, surfaces wetted by the nucleus, container walls, already formed crystal surfaces, etc.) can enhance the formation of stable embryos and have important implications for polymer crystallization [1]. Nucleating agents (NAs) are sometimes voluntarily added to the polymers in order to speed up processing times or improve their final properties. In most of the published works, the efficiency of different nucleating agents (NAs) is measured by empirical or qualitative methods and, therefore, is dependent on the adopted experimental conditions. For instance, a qualitative method consists of the observation of the morphology of crystallizing polymers induced by different substrates; a spherulitic morphology is representative of a low nucleation efficiency, while a transcrystalline one is characteristic of highly efficient nucleating surfaces [2]. Some widely employed quantitative empirical methods are the evaluation of the increase of crystallization temperature obtained in non-isothermal conditions, or the measurement of the reduction of the crystallization half-time in isothermal crystallization experiments [3,4,5], and the calculation of the ratio between the nucleation density at the substrate–polymer melt interface and the one in the polymer bulk, which is due to random heterogeneities active at a certain crystallization temperatures (the higher the nucleation ability of the substrate, the higher the calculated ratio) [6,7].

The above cited quantitative methods, due to their empirical nature, present limitations for the quantification of the intrinsic ability of a substance to enhance the crystallization of a specific polymer. For example, in the first two methods, limitations arise from the dependence on the NA concentration, on the cooling rate in non-isothermal experiments, and on the crystallization temperature in isothermal experiments. The third method cannot be used when the nuclei are not countable (i.e., due to high nucleation density) and still suffers from the dependence on experimental parameters such as crystallization temperature and observation time. Thus, in order to derive a universal nucleating efficiency scale of different substances for a certain semi-crystalline polymer, none of the above described methods can be employed. To this aim, a parameter independent from experimental conditions and representative of the intrinsic nucleating efficiency of the substance for a certain polymer is needed.

**Figure 1:**
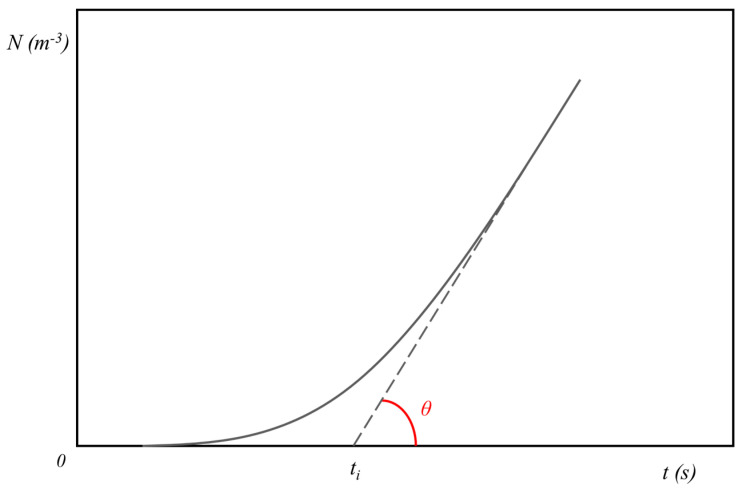
Schematic curve of the number of the nuclei density (*N*) as a function of time. *t_i_* is the induction time and the tangent of *θ* is equal to the nucleation rate in the steady-state (*I_st_*).

In the theory of heterogeneous nucleation, the correlation between the surface tension properties of the substrate, the polymer crystal, and the polymer melt is described by the interfacial free energy difference function, Δ*σ*. This parameter is equal to the difference in the system surface’s free energy resulting from the formation of the first crystalline layer onto the heterogeneous substrate, which practically substitutes a substrate/melt interface with a substrate/crystal and a crystal/melt interface. This difference is thus an intrinsic characteristic of the polymer/nucleating substance pair, independent from experimental conditions and thus suitable for comparing the nucleating ability of different substrates. The classical approach to calculate Δ*σ* consists of the determination of the nucleation rate, by measuring the density of nuclei as a function of time, during crystallization in isothermal conditions. However, this method is not suitable when the nucleating efficiency of the substrate is high, since a transcrystalline layer (TCL) forms at the interface with the nucleating substrate, and it is not possible to count the nuclei. A solution to this limitation, firstly proposed by Ishida and Bussi [8,9], consists of using the induction time (*t_i_*) as a measurable parameter. According to classical nucleation theory [10,11], the nucleation process can be separated into a transient-state and a subsequent steady-state. In the former, nuclei form and either dissolve again or grow up stochastically, while in the latter, the size distribution of new subcritical and critical nuclei is stationary, and a time-independent steady-state nucleation rate (*I_st_*) can be defined as the number of supercritical nuclei formed per unit time in a unit volume of the system. Indeed, the transient-state represents a time period needed for the evolution of the initial nuclei size distribution towards the stationary distribution established at the employed crystallization temperature, which results in a variation of the nucleation rate until it assumes the steady-state value. Eventually, by plotting the density of nuclei (number of supercritical nuclei per unit volume (*N*)) as a function of time, a linear segment is obtained, from which *I_st_* and the induction time (*t_i_*) can be derived as the slope and the intercept with the time-axis, respectively (see Figure 1). Thus, given this graphical definition, the induction time represents the time needed for the beginning of the steady-state nucleation and, therefore, an approximation of the time needed for the formation of stable (supercritical) crystal nucleus.

The concepts described so far are valid also for studying the nucleation process occurring in semi-crystalline polymers, in which the critical nucleus is represented by one or more layers of crystallizable segments of the polymer chains on the surface of the foreign substrate. In the work of Ishida and Bussi, as well as other investigations on fiber–polymer composite systems [12,13,14], the induction time was employed to calculate Δ*σ* by applying a model based on the assumption that the critical nucleus is completed when the first crystalline layer is deposited on the substrate surface. In the present investigation, the same method was applied for the first time to two NA–polymer systems widely used in industrial production and a fiber–polymer composite to show the applicability of the method to different kinds of nucleating substrates. The purpose was to achieve a quantitative differentiation of the intrinsic nucleating efficiency of these additives for a chosen semi-crystalline polymer, i.e., isotactic polypropylene (iPP).

Next to the “first layer model”, a more comprehensive approach, introduced by Muchova et al. [15,16,17], has also been tested on the obtained experimental data. In order to simplify the complicated nucleation process of polymers, Muchova distinguished two qualitatively different steps: the formation of the first layer of crystalline segments on the foreign substrate, which occurs within time *t_h_*, and the formation of further layers until the growth of the critical nucleus is completed, which occurs within time *t_s_*. Thus, the induction time is given by the sum of these two contributions:

(1) $$t_{i}=t_{h}+t_{s}$$

The two components of the induction time can be expressed by these relations:

(2) $$t_{h}=A_{1}\;exp\left(\frac{16\sigma \sigma_{e}\Delta \sigma {\left(T_{m}^{0}\right)}^{2}}{kT{\left(\Delta h^{0}f\Delta T\right)}^{2}}\right)\;exp\left(\frac{U^{*}}{R\left(T-T_{\infty }\right)}\right)$$

(3) $$t_{s}=A_{2}\left(\frac{2\Delta \sigma T_{m}^{0}}{\Delta h^{0}\Delta Tb_{0}}-1\right)\;exp\left(\frac{4\sigma \sigma_{e}b_{0}T_{m}^{0}}{kT\Delta h^{0}f\Delta T}\right)\;exp\left(\frac{U^{*}}{R\left(T-T_{\infty }\right)}\right)$$

where *A_1_* and *A_2_* are proportionality constants, *σ* is the free energy of the lateral surfaces in contact with the supercooled melt, *σ_e_* is the free energy of the surfaces perpendicular to the chain direction, Δ*h^0^* is the enthalpy of crystallization at the equilibrium melting point (*T°_m_*), *k* is the Boltzmann’s constant, *T* is the crystallization temperature, Δ*T* is the degree of supercooling (Δ*T = T°_m_ − T*), *f* is a correcting factor equal to *2T/(T + T°_m_)*, *b_0_* is the thickness of each newly formed layer, U* is the activation energy related to the transport of chain segments across the phase boundary, *R* is the gas constant and *T_∞_* is the temperature below which all motions associated with viscous flow cease.

The model previously applied in fiber–polymer composite literature was basically equivalent to the linearization of Equation (2):

(4) $$\ln\left(\frac{1}{t_{i}}\right)+\frac{U^{*}}{R\left(T-T_{\infty }\right)}=\ln\left(\frac{1}{A_{1}}\right)-\frac{16\sigma \sigma_{e}\Delta \sigma {\left(T_{m}^{0}\right)}^{2}}{k\Delta h^{o}{}^{2}}\frac{1}{T{\left(\Delta Tf\right)}^{2}}$$

Indeed, by plotting ln(1/t_i_) + U*/(R (T − T_∞_)) vs. *1/(T (*Δ*T f)^2^)*, it is possible to calculate the product *σσ_e_*Δ*σ* from the slope of the fitting line and in turn, knowing *σσ_e_* by growth rate measurement (according to the Hoffmann–Lauritzen theory) [18,19], Δ*σ* can be calculated. 

On the contrary, the model that considers the formation of further layers is based on a simplification and linearization of Equation (3) [16]:

(5) $$\ln\left(t_{i}\Delta T\right)-\frac{U^{*}}{R\left(T-T_{\infty }\right)}=\ln\left(\frac{2A_{2}\Delta \sigma T_{m}^{0}}{\Delta h^{0}b_{0}}\right)+\frac{4\sigma \sigma_{e}b_{0}T_{m}^{0}}{k\Delta h^{0}}\frac{1}{Tf\Delta T}$$

By plotting ln(t_i_ ΔT) − U*/(R (T − T_∞_)) vs. *1/(T f* Δ*T)*, Δ*σ* is included in the intercept of the fitting line with the y-axis.

In the first part of this work, the method used to experimentally derive the induction time for the various substrates is described. The application of the above cited induction time models to the experimental data will then be discussed in the second part of this paper, allowing us to make considerations regarding the determining step for the nucleation process in NA–polymer systems, and to calculate Δ*σ* for each substrate.

## 2. Materials and Methods 

A commercial, highly stereoregular Ziegler-Natta isotactic polypropylene (iPP) homopolymer received from Borealis Polyolefine GmbH (tradename HD601CF) was used throughout this work. The average molecular weight (M_w_) and polydispersity index (M_w_/M_n_) are 365 kg/mol and 5.4, respectively. The peak melting temperature, measured by differential scanning calorimetry (DSC) at a heating rate of 10 °C/min, is 164 °C. The iPP used in this work shows typical polydispersity for this class of polymers. Furthermore, the commonly adopted heterogeneous nucleation models for semi-crystalline polymers, such as the one used in this work, do not take into account polydispersity or molecular weight effects. In fact, the free energy barrier is independent of molecular weight since typical nucleus sizes are considerably smaller than the polymer random coil size. Therefore, any possible molecular segregation effect is typically of importance only for very low molar masses.

The investigated nucleating agents were sodium benzoate (NaBz), purchased from Sigma-Aldrich, and an organophosphate salt (sodium 2,2’-methylene *bis*-(4,6-di-*tert*-butylphenyl)phosphate), commercially named NA-11, kindly provided by Borealis Polyolefine GmbH. Both NAs promote the formation of the α-crystalline phase of iPP. In addition, the nucleation ability of a glass fiber (GF), a material commonly used in iPP fiber composites, was also studied for the sake of comparison.

The sample preparation consisted of obtaining thin iPP films (30–50 µm) by compression molding of the polymer between two pieces of aluminum foil on a heating stage set at about 200 °C in order to delete any memory in the melt [20], followed by the preparation of the nucleating substrates. Since the two NAs have a melting point exceeding 400 °C [21,22], compact aggregates of crystals, suitable as substrates for subsequent iPP crystallization, were prepared by precipitation from solution. A 7.5 wt% solution of NaBz in deionized water was drop cast on a microscope glass slide, and the solvent was evaporated at 60 °C to obtain a polycrystalline film. The film was then shaped with a wet blade to form a sharp linear interface of appropriate thickness. NA-11 was solubilized in methanol at a concentration of 0.5 g/100 mL and drop cast in a small region of a glass slide circumscribed by adhesive tape, in order to avoid excessive spreading of the drop. The evaporation of the solvent was carried out at 50 °C. Thermogravimetric and DSC analysis revealed the presence of some residual methanol, which was completely removed by a heat treatment at 220 °C for 20 min. After annealing, the brittle NA-11 substrate was broken into small flakes and transferred onto another clean glass slide. Finally, the iPP thin film was placed on top of the NA substrates and melted while gently pressing with a cover glass to ensure the contact between polymer and NA. Finally, the iPP/single-GF composite was prepared by placing a short piece of GF on a microscope glass slide, covering it with the iPP film, and subsequently melting the assembly covered by a second thin glass slide.

Subsequently, the prepared samples were isothermally crystallized inside of a Mettler FP90 hot stage coupled with a Leica DM-LP 100 microscope with crossed polarizers in order to observe the phase transition of the polymer. The stage temperature was calibrated by observing the melting of standard substances (benzophenone, benzoic acid and caffeine). For polymer crystallization experiments, the sample was first heated from room temperature to 200 °C and annealed for 3 min to erase any possible self-nucleation effect on subsequent cooling to the crystallization temperature. Then, the system was cooled at a rate of 20 °C/min to the chosen crystallization temperature, and kept in isothermal conditions for an adequate amount of time while the crystallization process was observed. An Optika B5 digital camera was fitted to the polarized optical microscope (POM) to capture images of the samples during the experiments. The recorded micrographs were subsequently analyzed with the image processing software ImageJ.

## 3. Results

**Figure 2:**
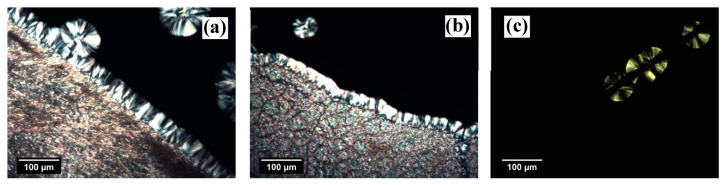
Morphologies obtained after isothermal crystallization at 142.5 °C for (**a**) sodium benzoate (NaBz), (**b**) sodium 2,2’-methylene *bis*-(4,6-di-*tert*-butylphenyl)phosphate (NA-11) and (**c**) glass fiber (GF). The crystallization times were: (**a**) 2.0 h, (**b**) 2.4 h and (**c**) 1.5 h.

The morphological evolution captured by POM during the isothermal crystallization of iPP at the interface with the two NAs and the GF is shown in Figure 2. In both NA–polymer systems, a clear transcrystalline layer (TCL) forms at the interface with the substrate. Since the density of nucleation sites is so high that impingements among the growing spherulites occur already a short time after the beginning of nucleation, the direction for further growth is only possible perpendicular to the interface. TCL formation testifies to the high nucleation ability of the substrates. In the micrographs relative to the NA–polymer systems, the formation of spherulites next to the TCL or in the bulk may also be due to the heterogeneous nucleation that occurred on either small pieces of NA that remained after the sample preparation or natural heterogeneities in the polymer. In the former case, the spherulites have a radius equal to the one of the spherulites nucleated at the interface with the NA, i.e., to the TCL thickness, since they started to grow at the same time, while in the latter case, since the NAs used are highly efficient, the spherulites nucleate later than the crystals at the interface and, therefore, their radius is smaller with respect to the TCL thickness.

On the contrary, in the GF–polymer system, the number of spherulites nucleated at the interface is easily discernable. This kind of nucleation mechanism is termed “sporadic” and is associated with a lower nucleation ability of the substrate.

**Figure 3:**
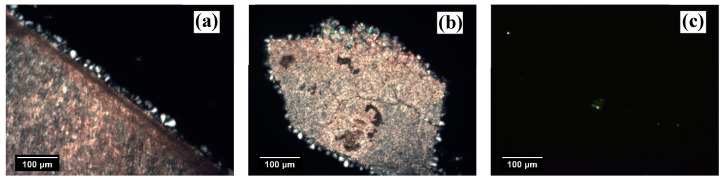
Morphologies obtained after isothermal crystallization at 152.5 °C for similar crystallization times (about 9 h) for (**a**) NaBz, (**b**) NA-11 and (**c**) GF.

At very high crystallization temperatures, the surface concentration of nuclei decreases due to the increased free energy barrier for the formation of a cluster of critical size. As shown in Figure 3a, upon isothermal crystallization at 152.5 °C, the number of spherulites nucleated at the interface with NaBz is lower than in the micrograph in Figure 2a, but still not easy to determine, since the achieved crystalline morphology is somewhere halfway between transcrystalline and sporadic. The same situation is observable for the iPP/NA-11 system after crystallization at the same temperature and time (Figure 3b). Therefore, given the high efficiency of the two NAs employed, it is not possible to detect differences in their nucleating ability solely by making qualitative observations on the obtained morphology. On the other hand, by increasing the crystallization temperature, it may be possible to obtain a clear sporadic nucleation at the interface and to determine the maximum temperature at which TCL can develop, which was suggested by Wang et al. [14] to be directly related to the nucleating ability of a given substrate. However, experiments at crystallization temperatures higher than 152.5 °C could not be performed because the iPP melt had a low viscosity and tended to flow away from the interface, providing unreliable results. Eventually, in the micrograph corresponding to the iPP/GF system (Figure 3c), the number of nucleation events that occurred on the surface of the GF decreased to one when the same low undercooling was applied. In this case, a very low undercooling is needed for achieving the TCL at the interface due to the rather low nucleating efficiency of this substrate for iPP.

Besides the qualitative observations so far described, a quantitative analysis of the recorded micrographs was performed. Given the transcrystalline morphology achieved in the NA–polymer systems, it was not possible to determine the nucleation rate directly by counting the spherulites nucleated at the interface, as the classical approach foresees. Therefore, the only suitable way to estimate Δ*σ* was the use of the induction time.

Since it was not possible to directly detect when the nucleation occurs from the micrographs obtained by POM, the calculation of *t_i_* was firstly attempted by using a method employed in a recent molecular dynamic simulation work [23], which will be referred to as the “extrapolation method”. However, the obtained induction time suffers from very poor reproducibility. Three sources of error have been identified: the rather large extrapolation of the data, the relatively low accuracy in the determination of the TCL growth front, and the approximation of the substrate–polymer interface (which is the most critical assumption).

On the contrary, the TCL growth rate can be measured with this method with good reliability as the slopes of the linear fitting lines of TCL thickness vs. time (see Figure S1 in the Supplementary Materials).

Therefore, given the issues with the above described method, the detection of the induction time has been attempted using the variation of the integrated intensity of polarized light through the sample during transcrystallization, a method called the “light intensity method”.

### Light Intensity Method

The method discussed in the following section has been inspired by Muchova et al. [15]. During isothermal crystallization, the increase of the light intensity passing through the sample with time, caused by the increase in the crystal phase content, can be recorded. Then, it is possible to unambiguously determine the time at which the light intensity starts to increase from its initial value, i.e., the induction time.

**Figure 4:**
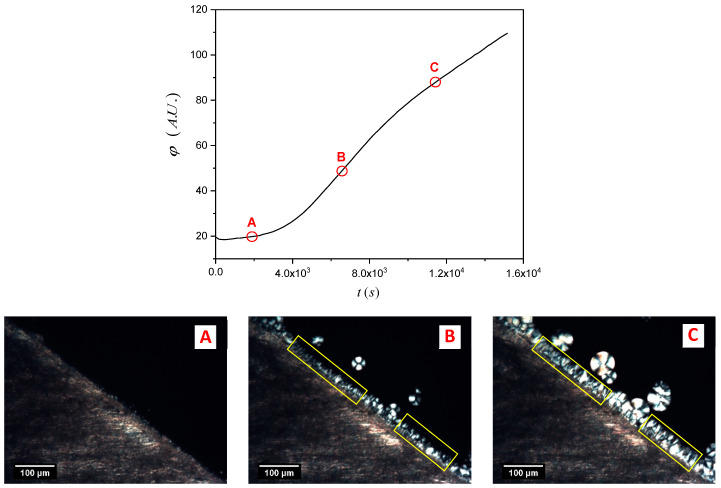
Mean gray value (*φ*) as a function of time during an isothermal experiment conducted at 147.5 °C for the isotactic polypropylene iPP/NaBz sample. Three micrographs, corresponding to the points A, B and C on the curve, are reported to show the morphological variation. The yellow rectangles indicate the region of interest used to compute the light intensity.

Once again, the software ImageJ was used to analyze the micrograph sequence recorded during the isothermal crystallization experiments. At first, a “region of interest” consisting of one or more areas within the picture where the TCL develops was selected, and then the mean gray value (*φ*) within the selected areas for every picture of the sequence was calculated by the software, as shown in Figure 4. The mean gray value is the sum of the gray values of all the pixels (after converting each pixel to grayscale) divided by the number of pixels in the selection. In the plot, an evident increase of the mean gray value in time, proportional to the growth of the TCL, can be appreciated.

The total area of the selection does not influence the measured onset time (*t_i_*). To better identify the induction time, the derivative of the mean gray value (rather than its absolute value) is used. This is very important since every sample has its characteristic morphological features (especially for the NA-11 substrates), and thus the selection of the integration region must be adapted to follow the particular interface shape, and to avoid the inclusion of spherulites nucleated away from the interface (see Figure 4).

According to Muchova, there are two possible relations between the derivative of the mean gray value with respect to time (or rate of luminous flux change) and time [15]:

(6) $$\frac{d\varphi }{dt}=KZ\nu^{4}{\left(t-t_{i}\right)}^{3}$$

(7) $$\frac{d\varphi }{dt}=KZ\nu^{2}\left(t-t_{i}\right)$$

where *ν* is the linear growth rate, which is constant at a given crystallization temperature, *Z* is the number of growing spherulites, *K* is a constant which involves the dependence of the luminous flux on the difference in the refractive indices of the ordinary and extraordinary beams for the birefringent crystal, *t* is the time and *t_i_* is the induction time. Equation (6) describes the time derivative of the luminous flux derivative when the spherulitic growth is three-dimensional (3D), while Equation (7) refers to the two-dimensional (2D) growth, which begins when the spherulite size becomes larger than the polymer film thickness. Equation (7) predicts a linear relation between the rate of luminous flux increase and the time, which can be easily distinguished in the experimental data.

**Figure 5:**
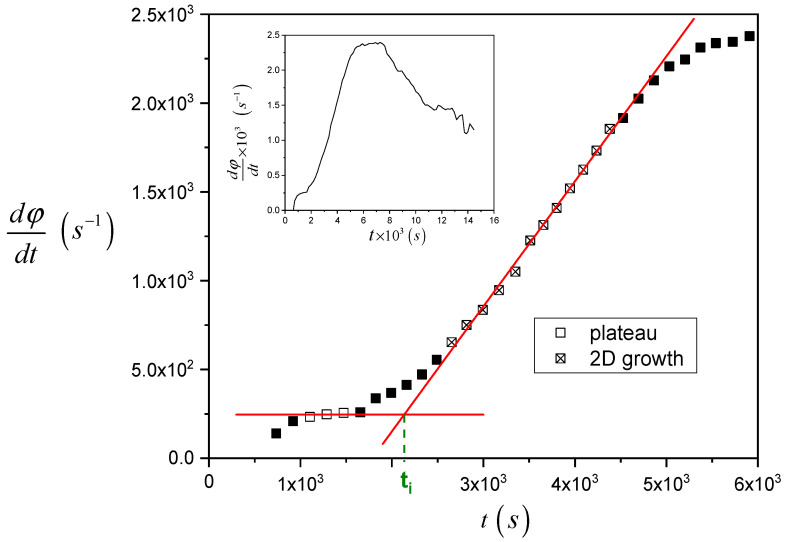
Example of the procedure for the calculation of *t_i_* (the raw data are shown in Figure 4). In the inset plot, the time-derivative of the mean gray value is shown over a more extended time range.

In Figure 5, the procedure to analyze the raw data and calculate the induction time is shown, taking as an example the data reported in Figure 4. The raw data are differentiated with respect to time and reported as a function of time. Two sets of data, i.e., before the nucleation starts (open square symbols, initial plateau) and when the two-dimensional growth of TCL is fully apparent (crossed open square symbols, linear increase region) are selected and fitted with linear regression. Finally, the *x*-coordinate of the intercept of the two fitting lines was considered as a measurement of the induction time.

The beginning of the 2D growth region in the plot of Figure 5 occurs after about 2700 s. On the other hand, from the data in Figure S1, the time needed for a spherulite crystallizing at this specific temperature (147.5 °C) to grow up to a size equal to the sample thickness (about 30 μm) would be about 14,300 s. Therefore, it can be deduced that growth becomes 2D even before that the spherulites in TCL have reached the size of the polymer film. This indicates that several spherulites are nucleated on the real interface of non-zero thickness. In this way, the growth of a given spherulite along the direction perpendicular to the substrate plane, after a certain time from its nucleation, is hindered by the impingement with other spherulites nucleated in sites located on the interface along the same direction (i.e., above or below the considered one), as well as in the direction parallel to the substrate plane.

**Figure 6:**
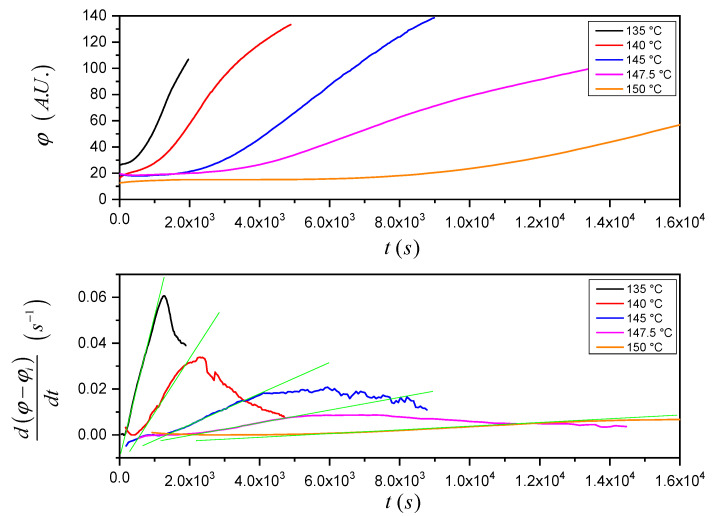
Mean gray value as a function of time recorded at the different indicated crystallization temperatures (**top**) and corresponding time-derivative of the normalized mean gray value vs. time (**bottom**). The green lines represent the linear fitting of the data in the two-dimensional growth region.

The reproducibility of the *t_i_* values obtained by means of this method is much higher with respect to the ones calculated by means of the previously described extrapolation method (see Figure S2 in the Supplementary Materials). As such, when experiments carried out at different crystallization temperatures are compared, reliable results can be achieved. Figure 6 (top) shows that the increase of the mean gray value with time is slower at higher crystallization temperatures. Furthermore, considering the time-derivative of the mean gray value (Figure 6 (bottom)) normalized to the initial mean gray value (*φ_i_*) for a better comparison of the curves, the parameters in Equation (7) (namely *ν^2^* (proportional to the slope of the green fitting lines) and *t_i_* (the intercepts of the fitting lines with the time-axis)) can be easily identified. The expected trends of *ν^2^* and *t_i_* with crystallization temperature are verified: with increasing crystallization temperature, the crystal growth rate becomes lower while the induction time gets longer.

An assessment of the reliability of the light intensity method can be obtained by comparing the square root of the slope of the 2D growth region fitting lines proportional to the linear growth rate (*ν*), with the growth rate measured from the TCL thickness evolution in time (*G*). A linear relationship between *G* and *ν* was indeed observed (see Figure S3 in the Supplementary Materials). Moreover, this result suggests that the temperature dependence of the number of growing spherulites *Z* (included in the *A* constant in Figure S3), is negligible in the explored crystallization temperature range. Apparently, this observation is in contrast with the micrographs shown in Figure 3. However, we note that among the investigated substrates, only NaBz underwent a consistent change in the crystallization morphology developed at the interface, i.e., from transcrystalline to sporadic, and only at the highest crystallization temperature. It is thus apparent that the change in the number of growing spherulites on the substrate has a smaller temperature dependence with respect to the induction time. Therefore, for the sake of simplicity in the calculation of *t_i_*, *Z* has been considered constant with respect to the crystallization temperature in the following data treatment. 

Thus, a sufficient confidence in the analysis of the light intensity arising from substrate-induced nucleation during iPP crystallization has been achieved. The application of this method to the determination of the induction time will be discussed in the next section.

## 4. Discussion

**Figure 7:**
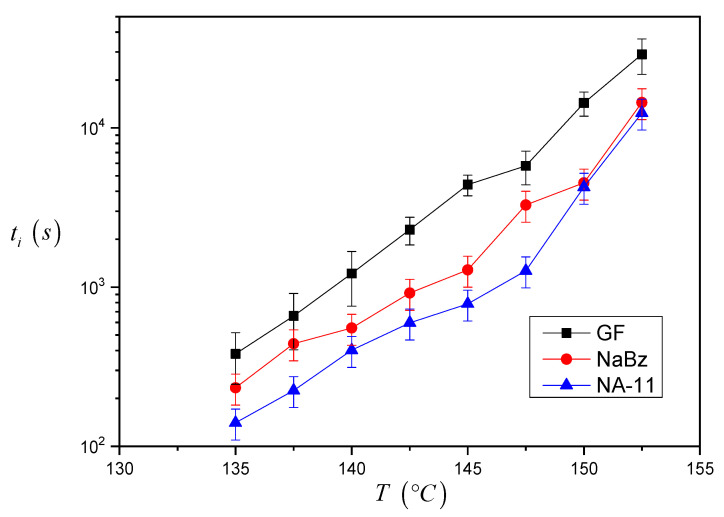
Induction time values at different crystallization temperatures for iPP in contact with GF, NaBz and NA-11 (the lines connecting the data of each series are just to guide the eyes). The data reported in the graph are the average of at least three repetitions and the average percent standard deviation is 20% (indicated by the error bars).

The induction time values calculated for the three systems are reported in Figure 7. As one can see, the *t_i_* values for the iPP/GF system are larger than the ones for the two iPP/NA systems, and thus the induction time seems to correctly reflect the expected nucleating efficiency of the substrate. Differences between the *t_i_* values of the two iPP/NA systems can also be appreciated and this must be related to the different nucleating ability of the two NAs under investigation: NA-11 has shorter induction times and therefore a higher efficiency than NaBz. At very high crystallization temperatures, it was not easy to complete the experiment since the iPP melt tended to flow away from the interface. For this reason, a smaller number of experiments were carried out at 150 °C and 152.5 °C, and the related data are considered less reliable.

From the results shown in Figure 7, it can be already ascertained that the induction time can be used to compare the nucleating efficiency of different substrates; however a model-based approach will be applied to calculate *∆σ* via the induction time and to determine a quantitative classification of the nucleating efficiency.

### 4.1. First Layer Model

This model, already adopted for fiber–polymer composite systems where a TCL forms at the interface between the two phases, assumes that the formation of the first layer of crystalline segments on the foreign substrate controls the overall nucleation rate, and it is described by Equation (2). Since a TCL at the substrate–polymer melt interface was also obtained in our case, an attempt to estimate *∆σ* for the three substrates by means of this model was initially made.

**Figure 8:**
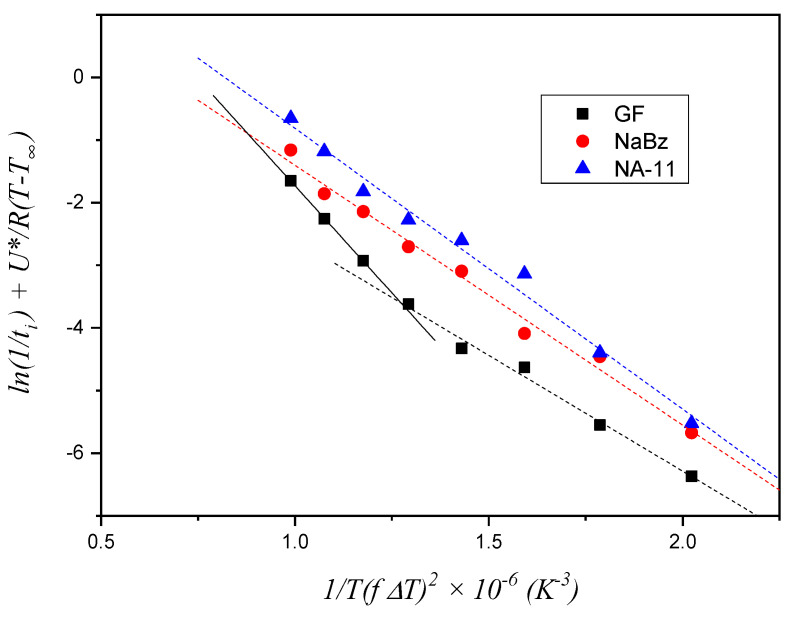
Plot of the experimental induction time data of the three systems according to the first layer model (Equation (2)). The constant values employed for U* and *T_∞_* are reported in Table 2, while the value of *T°_m_*, equal to 188 °C (461.15 K), was obtained from the best fit of the data.

The experimental values of *t_i_* for the three systems, shown in Figure 7, were employed to construct the plot in Figure 8. The Δ*σ* value is derived from the slope of the fitting lines, as stated in Equation (4). For the two iPP/NA systems, the fitting operation is performed on the entire temperature range investigated, while for the iPP/GF system, two distinct trends can be identified and, therefore, two fitting lines are drawn, fitting the data in the low and high temperature ranges, respectively. The values of the slopes are reported in Table 1.

Given that Δ*σ* is proportional to the slope of the fitting lines, the results shown in Table 1 do not conform to expectations. Indeed, according to this model, the small difference between the two NAs, would predict that NaBz is more efficient than NA-11 and that GF (at high temperatures) would be more efficient than both NAs. Clearly this conclusion is the opposite of what is shown by the experimental data of induction time. On the other hand, the fitting line for the GF data at low temperatures has a distinctly different slope with respect to all the others, which suggests that, with decreasing the temperature, the rate-controlling step of the nucleation process may change.

Thus, recalling the nucleation steps introduced by Muchova and briefly discussed in the introduction section, it can be hypothesized that in the temperature range used in these experiments, a transition between the two mechanisms (first layer/further layer controlling the nucleation rate) only occurs for the iPP/GF system, which has the lowest nucleating efficiency. For the other two highly efficient substrates, only one step is visible, and it should reasonably be attributed to the rate-control by time for the formation of further layers up to the attainment of a nucleus of viable size. In conclusion, the first layer model does not correctly account for the differences in the induction times observed in Figure 7.

As such, the model based on Equation (3) is used for the data analysis in the following section.

### 4.2. Further Layers Model

**Figure 9:**
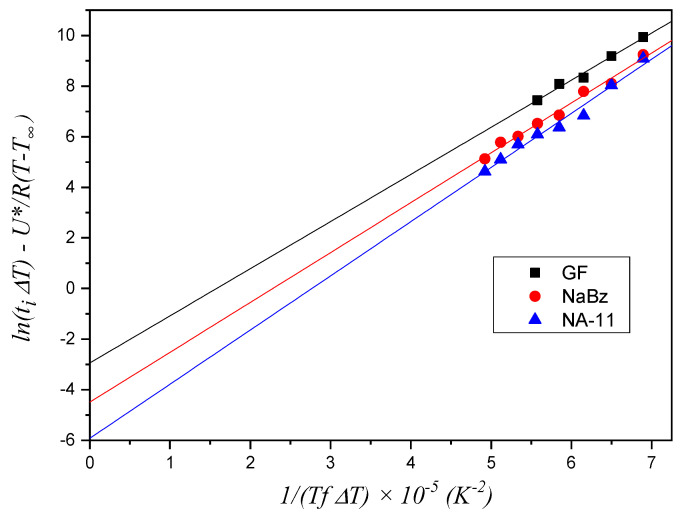
Plot of the experimental induction time data of the three systems according to the further layers method (Equation (3)). The constant values employed for U* and *T_∞_* are reported in Table 2, while the value of *T°_m_*, equal to 188 °C (461.15 K), was obtained from the best fit of the data.

The results of the application of the model described by Equation (3) to the induction time data are shown in Figure 9. For the iPP/GF system, only the data in the high temperature range were considered because, as seen in Figure 8, only these data presumably do not follow the first layer model. A meaningful difference in the values of the fitting line intercepts with the *y*-axis can be appreciated for the three systems, while the slope values are very similar, since they are exclusively dependent on the crystallizing material according to the model. The fitted values of slopes and intercepts are reported in Table 3. It can be seen that this model correctly captures the differences seen in the induction time of the three systems, attributing them to differences in the substrate nucleating efficiency (i.e., nucleation energy barrier).

This result leads to the conclusion that, for a quantitative analysis of the nucleation process, both models must be considered since each of them may be valid in a characteristic range of temperature, which depends on the substrate under study.

Although the further layers model can in principle be used to quantitatively compare the nucleating efficiency of the three systems, the obtained intercept values cannot be employed for the explicit calculation of each Δ*σ*, because the parameter *A_2_*, shown in Equation (5), is not experimentally accessible.

For this reason, an attempt to find the Δ*σ* values by fitting the experimental data with a detailed model, where the induction time is given by the sum of the individual times for the two steps (first layer/further layers), was made. In this way, the values for *A_1_*, *A_2_* (equal for the three systems) and for Δ*σ* (specific for each considered substrate) are obtained from the best fitting of the measured data.

### 4.3. Detailed Model Fitting

The equation describing the induction time in the detailed model was given by the sum of Equations (2) and (3), and can be simplified as:

(8) $$\begin{array}{cc} t_{TOT}=A_{1}\;exp & \left(\frac{16\sigma \sigma_{e}\Delta \sigma {\left(T_{m}^{0}\right)}^{2}}{kT{\left(\Delta h^{0}f\Delta T\right)}^{2}}\right)\;exp\left(\frac{U^{*}}{R\left(T-T_{\infty }\right)}\right)\\ & +A_{2}\left(\frac{2\Delta \sigma T_{m}^{0}}{\Delta h^{0}\Delta Tb_{0}}\right)\;exp\left(\frac{4\sigma \sigma_{e}b_{0}T_{m}^{0}}{kT\Delta h^{0}f\Delta T}\right)\;exp\left(\frac{U^{*}}{R\left(T-T_{\infty }\right)}\right) \end{array}$$

**Figure 10:**
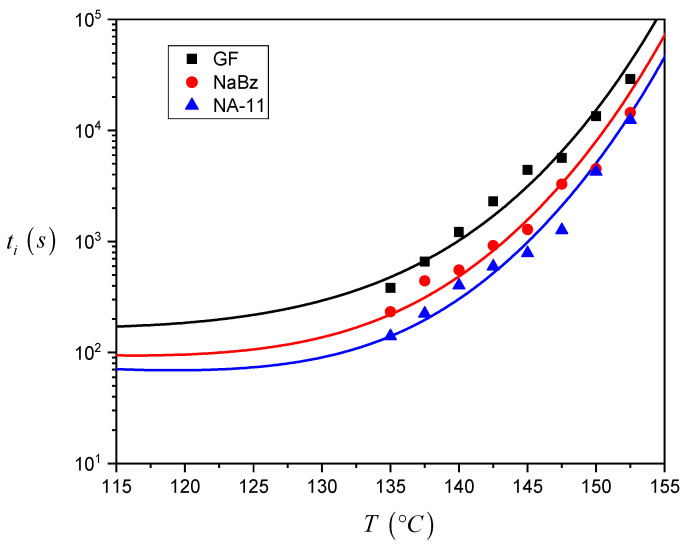
Plot of the induction time data as a function of crystallization temperature for the three systems, with the best fitting functions represented by the continuous lines and obtained through the simultaneous fitting of the detailed model (Equation (8)) on all experimental data.

The fitting operation has been done using Equation (8) in order to find the values of the five unknown parameters (*A_1_*, *A_2_*, Δ*σ_GF_*, Δ*σ_NaBz_*, Δ*σ_NA-11_*) such that a best fitting of the experimental induction time data for all the three systems was simultaneously obtained. The values of the constant parameters for iPP present in Equation (8) are reported in Table 2. The values found for these parameters are reported in Table 4 and the functions which fit the experimental data are shown in Figure 10. The Δ*σ* values obtained for the three systems agree with the experimental trend of the induction time data and with the expected nucleating efficiency scale.

**Figure 11:**
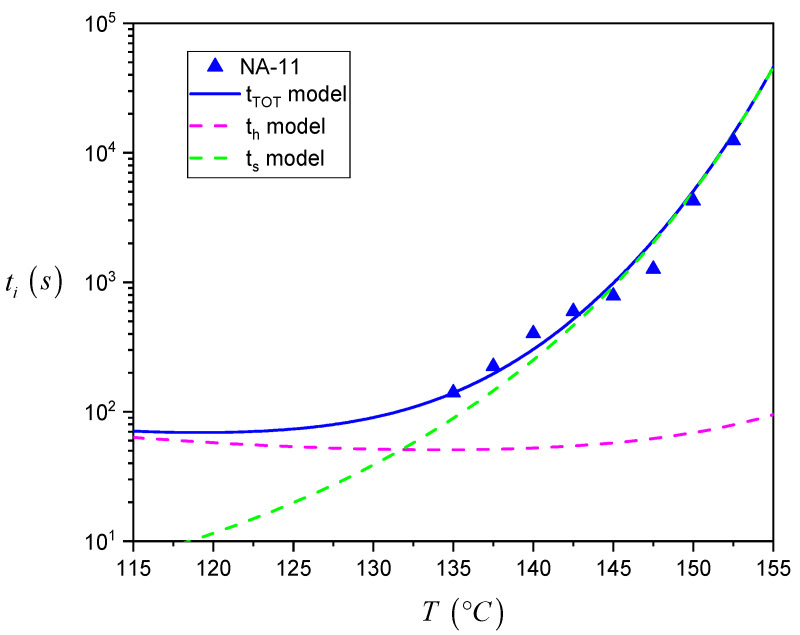
Plot of the experimental induction time data at different crystallization temperatures for the iPP/NA-11 system, together with the functions obtained from the fitting of the detailed model for the induction time (*t_tot_*). The dashed curves represent the contributions of the first layer and further layers formation (*t_h_* and *t_s_* models, respectively).

The description of the total induction time by the detailed model allows us to estimate in which range of temperature each of the steps proposed by Muchova is dominant and what their exact contribution to the overall induction time is. In fact, using the parameters reported in Table 1, it is possible to separately plot Equations (2) and (3) in the same plot. Figure 11 summarizes this analysis for NA-11, while the data for all the different systems can be seen in Figure S4 of the Supplementary Materials.

As one can see, at higher temperatures, *t_s_* is far larger than *t_h_*. As a result, the experimental data follow very closely the trend described by the *t_s_* model alone, i.e., the rate determining step in the nucleation process is the growth of the nucleus up to the critical size by addition of several crystalline layers. On the contrary, in the low crystallization temperature range, the time required to form the first crystalline layer of the nucleus becomes comparable to the one for its subsequent growth. As such, the data trend cannot be exactly estimated by using the *t_s_* model only, but is captured by the detailed model that correctly takes into account *t_h_*. Eventually, at very high supercooling temperatures which could not be explored in the present experiments, the overall induction time will be dominated by the formation of the first layer of crystalline stems on the substrate surface.

At this stage, it seems meaningful to compare the values of Δσ for the various substrates obtained by fitting the induction time (Table 3) with similar values for the same polymer derived from other literature studies. Wang et al. evaluated the interfacial free energy difference for the nucleation of polypropylene on a variety of fibers [25]. The values ranged from roughly 15 erg·cm^−2^ for low efficiency syndiotactic polystyrene fibers, to about 5 erg·cm^−2^ for the highly nucleating poly(tetrafluoroethylene). Clearly the value that we derived for the glass fiber is lower than any of the reported fibers. This result is inconsistent with the relatively low nucleating efficiency exhibited by GF, but it can be explained by considering the fact that the values from Wang et al. were all obtained by applying a heterogeneous nucleation model that only accounted for the formation of the first crystalline layer onto the substrate as the rate-determining step of the nucleation process. 

By comparing the values obtained for GF and NAs in this work, a three-fold decrease in Δ*σ* is appreciated. Due to the direct proportionality between this parameter and the free energy barrier for heterogeneous nucleation, the nucleation rate of iPP on NA-11 can be estimated as 20 times faster with respect to the one on glass fibers.

In our investigation, a very low value of Δ*σ* (0.7 erg·cm^−2^) was obtained for NA-11. Comparable values, lower than 1 erg·cm^−2^, are reported in the literature by Ishida and Bussi for the nucleation of a poly(caprolactone) (PCL) matrix onto polyethylene (PE) fibers [8] and for PE melt onto ultra-high molecular weight PE fibers [9]. In both systems, an epitaxial nucleation mechanism was inferred. In other investigations [26,27], the epitaxial crystallization between NA-11 and iPP was observed and explained on the basis of the matching between the lattice constant *b* of NA-11 and the *c* constant of the iPP monoclinic cell. As such, the obtained Δ*σ_NA-11_* is consistent with other cases of surface nucleation where epitaxial relationships between the two substances exist.

Urushihara et al. [26] determined the values of Δ*σ/σ* for iPP/NA-11 and iPP/NaBz, the same nucleating agents used in the present work. On the basis of the *σ* value indicated by the authors, Δ*σ* for the two systems is equal to 1.49 erg·cm^−2^ and 2.64 erg·cm^−2^, respectively. The absolute values of Urushihara are different from the presently determined ones, again due to the different model used for their determination. Nevertheless, the ratio between the Δ*σ* of iPP/NA-11 and iPP/NaBz derived from the Urushihara data is very close to the one established in this work, thus providing a certain confidence in the proposed method.

## 5. Conclusions

Crystallization of iPP on different substrates was investigated in order to study the heterogeneous nucleation kinetic and to propose a nucleating efficiency scale based on the surface free energy difference, Δ*σ*. The induction time was used as the experimental parameter to calculate Δ*σ* from isothermal crystallization experiments carried out with a POM, and analysis of the related evolution of transmitted light intensity. Through this method, meaningful differences in the induction times measured at different crystallization temperatures for three systems (iPP/NaBz, iPP/NA-11, iPP/GF) were found, confirming that the induction time is suitable for classifying the nucleating ability of different substrates.

By using the experimental induction time data, the calculation of *∆σ* was attempted through the application of two theoretical models proposed in the literature which identify either the formation of the first crystalline layer, or that of further layers until the attainment of supercritical dimensions as the rate determining step of the nucleation process. The first layer model does not result in meaningful differences among the *∆σ* of the three systems, or even worse, the deduced differences are not in agreement with the trend of the experimental data. On the contrary, the further layers model captures meaningful differences among the substrates, which are in agreement with the experimental data trend. A detailed analysis of the induction time model revealed that the first layer model is not incorrect, but rather not applicable to study the different nucleating efficiencies of the chosen substrates in the used temperature range. In fact, because the NAs are high efficiency substrates, the deposition of the first layer takes place in a very short time, which may even be an irrelevant contribution to the total induction time.

Nevertheless, the further layers model does not allow the direct determination of the absolute value of *∆σ* due to the presence of some unknown parameters. Therefore, a detailed model, given by the sum of the equations of the two said elementary step times, was used for the fitting of the experimental dataset. The values of *∆σ* obtained in this way are thus meaningful and reflect the expected nucleating efficiency scale of the three substrates.

## Figures and Tables

**Table 1:** Slope values of the fitting lines reported in Figure 8.

Substrate	Slope (× 10^6^) (K^3^)
GF (low T)	6.84 ± 0.08
GF (high T)	3.70 ± 0.22
NaBz	4.15 ± 0.18
NA-11	4.49 ± 0.21

**Table 2:** Values of iPP parameters employed for the fitting operations.

Parameter	Value
^a^ *σσe*	1.79 erg^4^·cm^−4^
^b^ *b0*	20.96 Å
^b^ Δ*h0*	1.96 × 10^9^ erg·cm^−3^
^c^ U*	6.28 × 10^10^ erg mol^−1^
^c^ *T∞*	232.15 K (−41 °C)

^a^ Calculated from growth rate measurements. ^b^ Reference [24]. ^c^ Reference [14].

**Table 3:** Slope and intercept values of the fitting lines in Figure 9.

Substrate	Slope (× 10^5^) (K^2^)	Intercept
GF	1.86 ± 0.13	−2.94 ± 0.82
NaBz	1.97 ± 0.09	−4.49 ± 0.53
NA-11	2.14 ± 0.13	−5.92 ± 0.76

**Table 4:** Parameters of Equation (8) which give the best fitting of the three series of experimental induction time data.

*A_1_* (s)	*A_2_* (s)	Δ*σ_GF_* (erg·cm^−2^)	Δ*σ_NaBz_* (erg·cm^−2^)	Δ*σ_NA-11_* (erg·cm^−2^)
0.314	1.14∙10^−4^	2.0	1.1	0.7

## Supplementary Materials

The following are available online at https://www.mdpi.com/1099-4300/21/11/1068/s1, Figure S1: Growth rate study by means of the extrapolation method, Figure S2: Reproducibility of the results obtained by means of the light intensity method, Figure S3: Assessment of the light intensity method, Figure S4: Single contributions to the total model.
