# Supplementary material for: Estimating the Nucleation Ability of Various Surfaces Towards Isotactic Polypropylene via Light Intensity Induction Time Measurements
Source: Entropy (Basel). 2019 Oct 31;21(11):1068. doi: 10.3390/e21111068 (PMC7514372; doi:10.3390/e21111068)
Supplement: Supplementary file 1 [file entropy-21-01068-s001.pdf]

entropy

MDPI

SUPPLEMENTARY MATERIALS

Extrapolation method

The method consists in measuring the thickness of the TCL in time and then extrapolating the regression line of the TCL thickness to its intersection with the time-axis, considered as the value of t_i. The measure of the TCL thickness was made by using the software ImageJ. For each experiment, a sequence of images captured at different time steps from the beginning of the isotherm was considered. In the software, the substrate-polymer melt interface was approximated by a straight line, and then about fifteen points on the TCL growth front were chosen for each picture. Finally, the average value of the TCL thickness at each time was obtained by averaging the fifteen point-line distances.

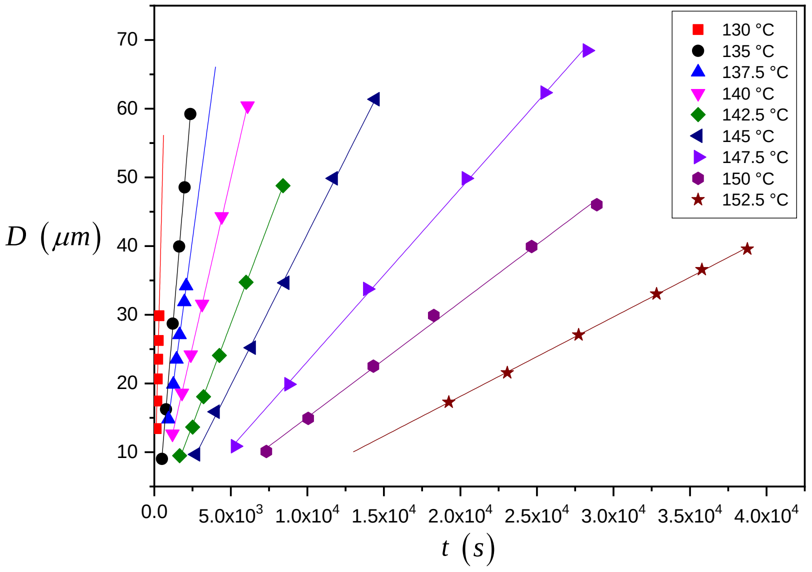

Figure S1. Growth rate determination by means of the extrapolation method. Thickness of the TCL (D) as a function of time, calculated for different crystallization temperatures (reported in the legend), using NaBz as substrate.

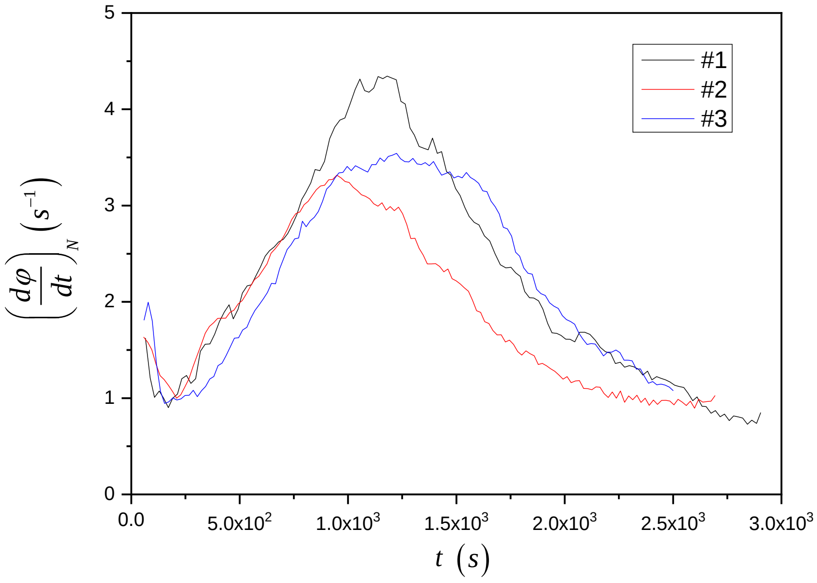

Figure S2. Reproducibility of the induction time results obtained by means of the light intensity method. The time-derivative of the mean gray value, normalized by the average mean gray value in the initial plateau region, is reported as a function of time for three iPP/NaBz samples crystallized at 135 °C. The induction time values are: $t_i(1) = 217$ s, $t_i(2) = 226$ s, $t_i(3) = 312$ s. The values of the slope in the 2D growth region are: *slope(1)* = 3.68*10^{-3} s^{-2}, *slope(2)* = 3.54*10^{-3} s^{-2}, *slope(3)* = 3.61*10^{-3} s^{-2}.

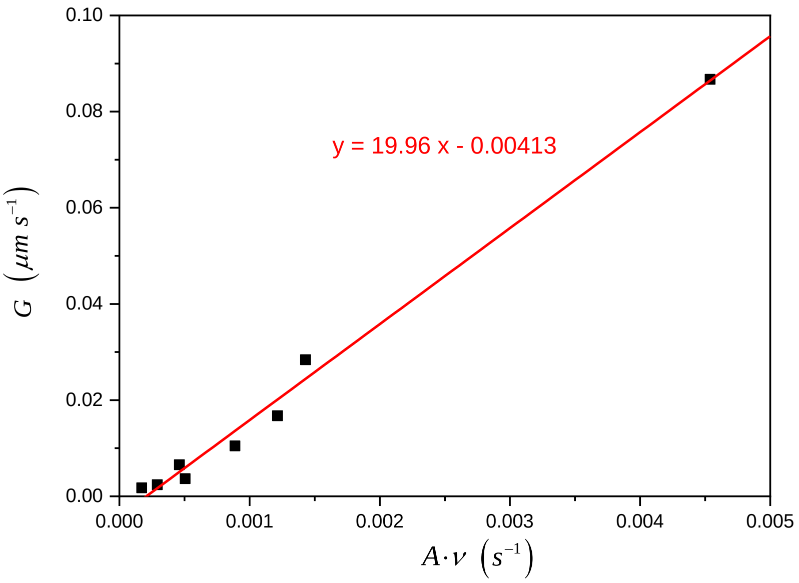

Figure S3. Assessment of the reliability of the light intensity method. Growth rate (*G*) values at different crystallization temperatures (from Figure S1) plotted as a function of the parameter *Aν*, obtained through the light intensity method, where *A* is the square root of *K* and *Z* (see Equation (7)). In the graph the equation of the fitting line is also reported.

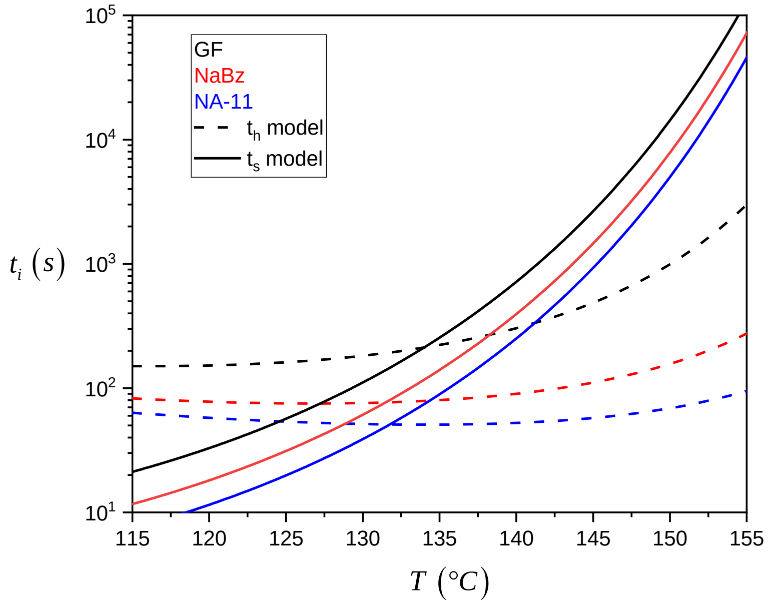

Figure S4. Contributions of first layer and further layers models to the overall induction time. Plot of the contributions of t_h and t_s models, derived from the fitting of the detailed model (Equation (8)) for each system.

© 2019 by the authors. Licensee MDPI, Basel, Switzerland. This article is an open access article distributed under the terms and conditions of the Creative Commons Attribution (CC BY) license (http://creativecommons.org/licenses/by/4.0/).
